# Expanding Gene-Editing Potential in Crop Improvement with Pangenomes

**DOI:** 10.3390/ijms23042276

**Published:** 2022-02-18

**Authors:** Cassandria G. Tay Fernandez, Benjamin J. Nestor, Monica F. Danilevicz, Jacob I. Marsh, Jakob Petereit, Philipp E. Bayer, Jacqueline Batley, David Edwards

**Affiliations:** School of Biological Sciences, The University of Western Australia, Perth, WA 6009, Australia; cassandria.tayfernandez@research.uwa.edu.au (C.G.T.F.); benjamin.nestor@research.uwa.edu.au (B.J.N.); monica.danilevicz@research.uwa.edu.au (M.F.D.); jacob.marsh@research.uwa.edu.au (J.I.M.); jakob.petereit@uwa.edu.au (J.P.); philipp.bayer@uwa.edu.au (P.E.B.); jacqueline.batley@uwa.edu.au (J.B.)

**Keywords:** pangenomes, CRISPR-Cas, structural variations, gene editing, genomes

## Abstract

Pangenomes aim to represent the complete repertoire of the genome diversity present within a species or cohort of species, capturing the genomic structural variance between individuals. This genomic information coupled with phenotypic data can be applied to identify genes and alleles involved with abiotic stress tolerance, disease resistance, and other desirable traits. The characterisation of novel structural variants from pangenomes can support genome editing approaches such as Clustered Regularly Interspaced Short Palindromic Repeats and CRISPR associated protein Cas (CRISPR-Cas), providing functional information on gene sequences and new target sites in variant-specific genes with increased efficiency. This review discusses the application of pangenomes in genome editing and crop improvement, focusing on the potential of pangenomes to accurately identify target genes for CRISPR-Cas editing of plant genomes while avoiding adverse off-target effects. We consider the limitations of applying CRISPR-Cas editing with pangenome references and potential solutions to overcome these limitations.

## 1. Introduction

The world’s population is predicted to increase to nearly 10 billion people by 2050 [1], coupled with a predicted increase of average surface temperature of 2 °C by 2043 [2] and more variable weather patterns. Hence, there is a need for more climate change-ready crops with increased yield [3,4]. Genome-editing technologies using nucleases such as Clustered Regularly Interspaced Short Palindromic Repeats and CRISPR associated protein Cas (CRISPR-Cas), Zinc finger nucleases (ZFNs) [5,6,7], and transcription activator-like effector-based nucleases (TALEN) [8,9,10,11] have already demonstrated their capacity in supporting crop resilience and yield improvements in several species [12,13,14,15,16], but the lack of knowledge of genome diversity and the polyploid nature of some crop genomes complicates the targeting of gene editing sites, leading to inefficiency in the traits that could be improved and the potential for adverse off-target effects from gene-editing experiments [12,17].

Modern crops have been subjected to breeding and selection, which often leads to large modifications in the genome and can reduce the efficiency of genome editing to improve specific functional traits or analyse traits through mutagenesis [18,19,20]. There is significant genome variation present between individuals, and this genomic variation can be associated with important functional traits [21,22,23,24,25,26], such as nucleotide deletions linked with embryo sac fertility and presence/absence variation of genes (PAV) linked with submergence tolerance, yield, and phosphorus deficiency tolerance in rice (*Oryza sativa*) [26]; PAV linked with silique length, seed weight, and flowering time in *Brassica napus* [27]; PAV associated with disease resistance, acyl lipid metabolism, and glucosinolate metabolism in *B. napus* [28]; and SNPs linked with number of branches, number of seeds per pod, number of pods per plant, plant height, seed weight, and seed yield in pigeon pea (*Cajanus cajan*) [29]. Gene editing to improve agronomically-significant traits is challenging if the target gene is not present in the reference genome sequence and the sequence cannot be used to tailor the genome editing experiment, and so single reference genomes are often inadequate for designing editing target sites.

The compilation of multiple genome sequences into a pangenome instead of a single reference genome provides a genomic sequence resource that can adequately represent genome diversity in different varieties or species. The advantages and disadvantages of CRISPR-Cas, TALENs, and ZFNs have been extensively reviewed, with CRISPR-Cas generally being easier to design and use and TALENs allowing higher specificity to targets [30,31,32,33], so here we focus on the CRISPR system for genome editing, including CRISPR/Cas9 and CRISPR/Cpf1 (previously known as CRISPR/Cas12a).

## 2. Pangenomes

Pangenomes were first introduced by Tettelin et al. [34] to describe gene diversity in *Streptococcus agalactiae*. Pangenomes can be constructed through the sequencing of individual genomes or survey of genetic variations within a species to describe the extensive repertoire of variation. The use of pangenomes removes the sample bias caused by using single reference genome assemblies, allowing the identification of structural variations (SVs) to assess the diversity within species. This diversity can encompass PAVs and non-genic regions. Genes in pangenomes can be classified as core, present in all individuals of the species, or dispensable, where they are absent in at least one individual, also known as accessory or variable genes. These gene classifications are sometimes extended to include private genes (present in 1% or fewer individuals) and near core/shell genes that are present in 99% or more of individuals [28,29,35,36,37].

**Figure 1:**
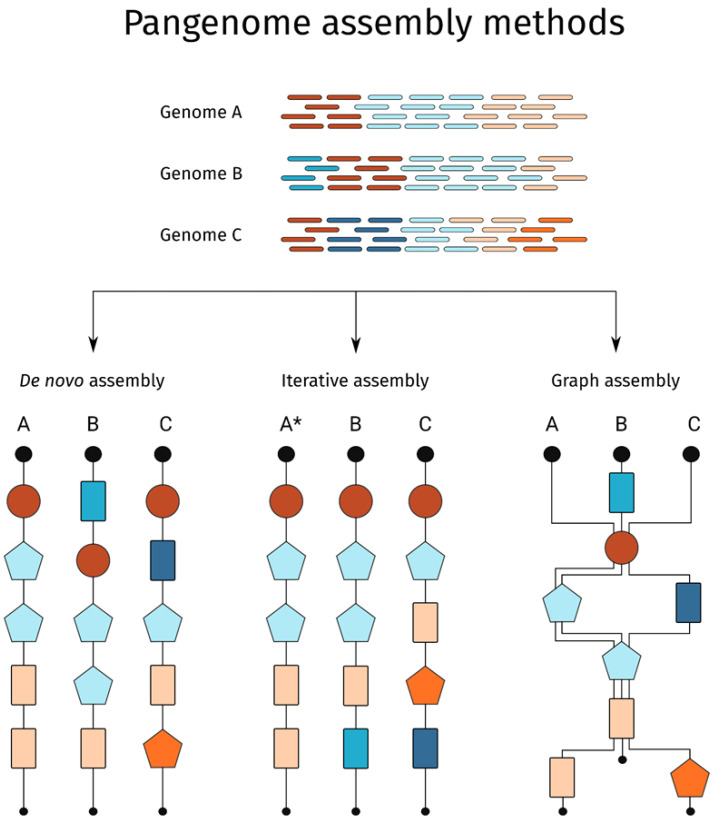
Diagram of pangenome construction methods based on genome sequencing data. Genome sequencing reads for genomes A, B and C are shown at the top of the image, each colour represents a gene in the genome. The genome sequencing reads may be assembled into a pangenome using de novo, iterative and graph-based assemblies which may influence the positioning of the assembled genes. The * indicates that genome A is used as reference genome in the iterative assembly method.

Pangenomes are mostly constructed in one of three ways (Figure 1). The first, de novo sequencing and comparison, involves the sequencing, assembly, and comparison of multiple genomes to identify core and variable genes and genomic regions [38]. This approach reveals the physical position of genes and other genomic elements. However, errors in assembly and annotation may lead to the false calling of variation [28]. Furthermore, this approach is costly and requires high-quality data with high sequencing coverage, limiting the application to relatively few individuals. The second method for pangenome construction, iterative mapping, and assembly, uses a single reference genome as a base for the pangenome. Whole genome sequence data for multiple individuals is aligned to the reference genome and any non-aligning sequence reads are assembled and added to the reference to build a pangenome [28]. This approach is less expensive than de novo assembly and comparison as it requires much less data, and so permits the assessment of large numbers of individuals with relatively low sequencing coverage. After pangenome construction, gene PAVs can be called by realigning the sequencing data from each individual back to the final assembled pangenome. This approach usually only calls PAV within genes and requires further analysis to accurately place the non-reference contigs within a genomic context. However, a combination of de novo assembly for a small number of representative individuals together with iterative assembly using large numbers of low coverage individuals provides both genomic context and PAV at a population scale, permitting in depth diversity analysis [34,39].

The third way to assemble pangenomes uses graph-based approaches, including sequence and variation graphs (VGs) [27,40] and practical haplotype graphs [41]. Pangenome graphs can be constructed from whole genome assemblies or by de novo graph genome assembly. Instead of a single representative sequence, the graph represents genomic variants as multiple paths. Sequence regions shared between individuals are collapsed into a single path and SVs are added to the graph as a node at the genomic location of their discovery [42,43]. In doing so, variant information for dispensable regions are stored as unique paths through the graph, displaying genomic diversity and sequence conservation [27]. Graph-based pangenomes can provide gene position information, but are computationally intensive to assemble, and graph quality correlates directly with the quality of the input data. However, with further advancements in DNA sequencing and data processing, particularly the expansion of high quality long read data, pangenome graphs will become the standard approach to assemble pangenomes. Regardless of the manner of construction, pangenomes can provide comprehensive data resources that can be used in trait association and in guiding the CRISPR-Cas design, supporting efficient genome editing.

Due to falling sequencing costs and the increased acknowledgment of significant gene presence/absence variation in some species, pangenomes have expanded beyond bacteria to higher organisms such as chicken [44] and human [45] as well as many plant species, allowing the analysis of the large-scale PAV observed in plants [46,47]. Pangenomics in plants was first proposed by Morgante et al. in 2007 [48] and since then, pangenomes have been assembled for many crop plant species including soybean (*Glycine max*) [49,50], maize (*Zea mays*) [51], tomato (*Solanum lycopersicum*) [35], *Brassica oleracea* [39], *Brassica napus* [27,52], *Brachypodium distachyon* [53], barley (*Hordeum vulgare*) [54], rice [55], pigeon pea (*Cajanus cajan*) [29,56], apple (*Malus domestica*) [57], capsicum [25], sesame (*Sesamum indicum*) [58], sunflower (*Helianthus annuus*) [59], yuca (*Manihot esculenta*) [60], sorghum (*Sorghum bicolor*) [36,61], and bread wheat (*Triticum aestivum*) [62]. Pangenomes for non-food plant species such as *Arabidopsis thaliana* [63], *Amborella trichopoda* [64], cotton (*Gossypium*) [65], and barrel clover (*Medicago truncatula*) [66] have also been published (Table 1). These pangenomes are valuable resources for studying genomic variation within plant species, assisting with the association of genes with traits, and supporting accurate and specific guide RNA design. For example, the use of pangenomes has identified genes corresponding with disease resistance gene analogs (RGAs) in *B. oleracea* [67] and disease resistance gene loss in cotton [65] that can be further targeted.

## 3. Association Analysis Using Pangenomes Can Reveal Valuable Sites for Genome Editing

**Figure 2:**
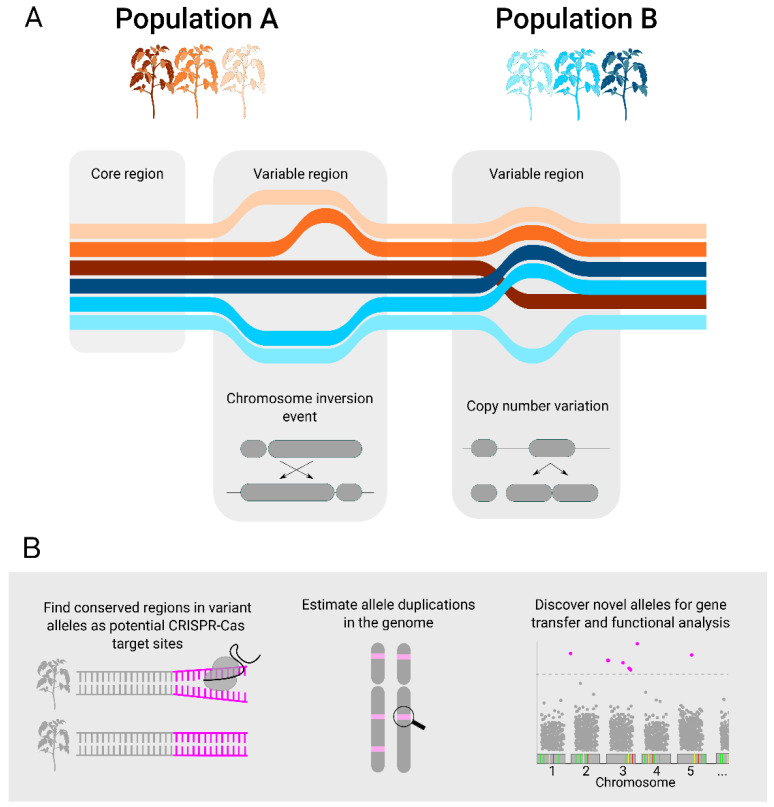
(**A**). Representation of a pangenome assembly composed of genomes from six individuals sourced from two populations. The core and variable regions of the pangenome are highlighted in this representation, in which the genetic diversity observed in the variable region can be caused by chromosome inversion or copy number variation (CNV). (**B**). Potential benefits of using pangenome reference for genetic modification, as the genetic diversity analysis can be used to define target sites in variant alleles, identify CNV that influence CRISPR-Cas mutation effectiveness and discover novel target alleles.

The regulation of gene expression and functional genome analysis using CRISPR-Cas systems has been widely demonstrated [37,69,70,71], including in the development of improved crops [71,72,73]. However, the editing efficiency achieved in plant studies can vary depending on the genotype and target site selected [72,73,74]. The inconsistency in CRISPR-Cas mutation rate can be partially attributed to target site GC content, target accessibility (due to chromatin state), and sgRNA secondary structure [37,69,75,76]. Successful editing can be even more challenging in polyploid plants because of the potential to edit multiple alleles or overcome genomic redundancy that may disguise the impact on the phenotype [77,78]. Detecting variant alleles and mapping their position in a pangenome allows for the design of allele-specific CRISPR sgRNA, as the alleles may have distinct effects in the plant phenotype. For example, the mlo gene discovered in barley (*Hordeum vulgare*) was used for decades in several crop species for inducing broad-spectrum resistance to powdery mildew. However, the pleiotropic effects of mlo can negatively affect yield [79]. To circumvent this issue, different mlo allele combinations can be used to modulate the degree of plant susceptibility to the pathogen and pleiotropic phenotype [80]. In wheat, mlo mutant plants also showed an allele-specific level of enhanced susceptibility to powdery mildew disease [79]. Numerous other examples of allele-specific phenotypes were observed to modulate crop disease resistance [59,81,82], abiotic stress tolerance [83], herbicide resistance [84,85], and yield in polyploid crops such as wheat [86] and camelina (*Camelina sativa*) [87]. In camelina, the selective mutagenesis of the three delta-12-desaturase genes (FAD2) showed reduced levels of polyunsaturated fatty acids and increased accumulation of oleic acid in the oil, corresponding with the different alleles for the three FAD2 loci [87]. The specificity of CRISPR-Cas opens new doors to testing the effects of individual small variants against a controlled genetic background. CRISPR-Cas can be used to study the effect of gene dosage by generating a series of allelic mutants through knock-out/down mutation of specific variant alleles [88,89]. A pangenome analysis associated with phenotypic information can assist the identification of these variant alleles and delimit CRISPR-Cas target sites, leading to the development of better performing varieties in the field. Structural variance uncovered by pangenomes can provide new alleles for genome functional analysis and also give detailed information about target allele location and accessibility in the genome (Figure 2).

**Figure 3:**
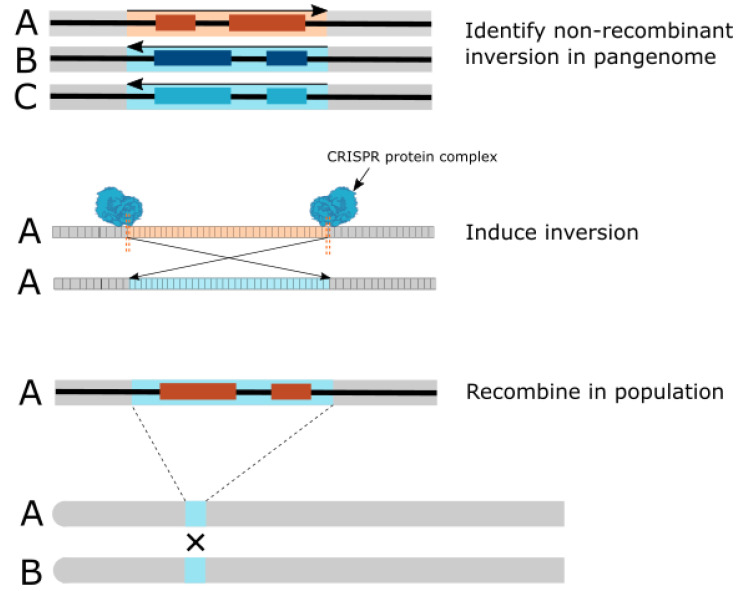
Reversal of inversion through CRISPR to allow crossing of inverted genes. A pangenome is used to identify a non-recombinant inversion in individual A compared to individuals B and C. CRISPR-Cas proteins are then used to induce double-stranded breaks at specific target sites in the inverted region, leading to re-inversion of the genomic segment and accessibility of the locked genes for recombination. The previously inverted genes in individual A can then be crossed with other individuals in the population.

A feature of pangenomes is the ability to show the impact of chromosomal inversions that can then be targeted by CRISPR-Cas editing for re-inversion (Figure 3). Chromosomal inversions can have a considerable impact on crop breeding as inverted regions are often prevented from crossing during recombination. In maize, one example of pangenomic analysis of chromosomal inversions is reported, using platinum-grade reference genomes from 66 maize key inbred lines. This analysis revealed several large (more than 100 kb) chromosomal rearrangements including insertions, deletions, and inversions on all 10 chromosomes, with the largest inversion spanning 75.5 Mb in the pericentric region of chromosome 2 [90]. The identification of this large structural variant by pangenome analysis and subsequent re-inversion of the genomic segment using CRISPR-Cas9 re-established the previous chromosomal state, allowing for the genes locked in this region to be accessed for recombination with the other inbred lines, which otherwise would be unfeasible [89]. This re-inversion in maize demonstrates the potential of pangenomes to identify chromosomal rearrangement boundaries with high precision and allows the editing of large regions of the chromosome within these chromosomal rearrangement boundaries using CRISPR-Cas.

## 4. Targeted Mutagenesis Guided by Pangenomes

Understanding the relationship between genotypes used in pangenome construction can assist in the identification of agronomically important SVs and subsequent targeted mutagenesis, particularly when comparing domesticated species to wild relatives. A recent study assembled a rice pangenome composed of 66 accessions that displayed green revolution phenotypes such as reduced height and early flowering phenotypes, with the aim of uncovering the underlying genes controlling these traits [91]. SV analysis of this rice pangenome identified 129 conserved gene loci potentially related to the shared phenotype. The analysis was followed by a subsequent CRISPR-Cas knock-out/down study uncovering 31 high yield-related genes, including six previously reported genes such as the sd1 semi-dwarf gene [91]. In a similar vein, the pangenome for medicinal cannabis (*Cannabis sativa*) was used to mine for cannabinoid biosynthesis genes, and 145 sgRNAs were generated for genes in the cannabinoid biosynthesis pathways [92]. These pedigree-based approaches for pangenome analysis take advantage of clear directional selection for finding conserved candidate genes among individuals with the desired phenotype, showing how pangenome-associated data can provide a powerful resource to uncover the role of genomic variations on the phenotype.

The inclusion of omics data such as transcriptome, metabolome, and proteomes can further support gene functional characterisation in pangenomes. A study *in Brassica napus* employed transcriptome-wide association studies in a constructed pangenome to identify QTLs related to regulating seed oil content in eight different environments across multiple years (2012–2018). Initially, 692 genes and four sets of coexpressed genes were significantly associated with seed oil content based on the seed transcriptome. A collection of genes more likely related to seed oil content were ranked using a gene prioritisation framework based on the multi-omics dataset. CRISPR-Cas9 and T-DNA mutants were employed to validate candidate gene function, revealing that two homologous genes (BnPMT6s) negatively regulate seed oil content [93]. Another study mapped 359 previously identified QTLs related to tomato flavour and aroma to the tomato pangenome, defining potential target regions for improving tomato lines [94]. The tomato pangenome was used to find promoter regions associated with QTLs related to tomato aroma, resulting in the identification of promoter alleles such as FLORAL4, which were present only in wild lines and have been lost during domestication [94]. These publications show that functional analysis of pangenome variable regions using omics data can broaden the understanding of QTL region conservation during domestication, assist in discovering structural variation and novel alleles, and map previously reported QTLs to support the selection of candidate genes for mutagenesis.

In silico association studies using small variants such as GWAS are usually limited to identifying a set of co-located, co-inherited loci linked within a haplotype [95]. However, in rice it was shown that up to 41.6% of trait-associated SNPs are located in presence/absence variable regions of the genome [96], which may be overlooked in a single reference genome. Pangenomes support comprehensive haplotyping by providing a genomic resource with variants across a diverse population of individuals, providing a full set of targets for modification with CRISPR-Cas [26]. In cases where the trait-associated haplotype is present in or near a gene, reverse genetics can be applied for inference of gene function through disruption of the promoter region with CRISPR-Cas [70,91]. However, traditional knockout experiments fail to characterise the specific effects of different small variants that could be contributing to a given trait of interest. Knockout experiments also rely on accurate gene annotations, which can be erroneous. Hence, characterising the individual and combinatorial effects of small variants linked within a trait-associated haplotype requires parallelizing sequence modifications at different genomic positions [89].

The first effective CRISPR-Cas toolkit for multiplexed editing in plants was developed in 2014 [97], though large-scale editing was not conducted until 2017 with the construction of mutant libraries for rice involving over 100,000 sites [98,99]. Since then, multiplexed editing has been applied to induce novel mutations in genic regions for rice, *Brassica napus* [100], soybean [101], and maize [37]. Whilst research with high throughput mutagenesis is promising, mutant libraries still do not functionally validate existing small variants present within and across plant populations. Small variant functional validation was accomplished in 2018 in humans using an approach called ‘saturation editing’, where CRISPR-Cas was used to assay 96.5% of all SNPs across 13 exons encoding for functional domains of the breast cancer susceptibility gene BRCA1 [102]. A potential solution to making small variant characterisation cost-effective in plants is to concentrate the established plant multiplexing methods to conduct saturation editing on specific trait-associated haplotypes from pangenomic datasets. Isolating the impact of specific small variants and allelic combinations identified in pangenomes will aid in elucidating the biochemical mechanisms involved in functional pathways. This understanding of variants and allelic combinations will provide a comprehensive understanding and precise control over specific variants underlying agronomic traits [101], enabling breeders to produce tailored crop varieties.

## 5. Off-Targets Effects in Multiplexed Editing

Off-target effects in CRISPR-Cas are often prevalent when employing multiplexed genome editing, particularly associated with assembly, expression, and processing of sgRNAs arrays and efficient delivery using current transformation methods. Multiplex genome editing involves simultaneously modifying multiple loci with multiple or single target-specific gRNA(s) [103]. The number of loci that can be edited by CRISPR-Cas in parallel is improving, but some technological challenges still remain in high-throughput mutagenesis. Beyond bottlenecks in throughput of sgRNA design and synthesis, multiplexing can lead to unintended interactions and competition between parallel CRISPR machinery [104,105] that can reduce binding specificity and efficiency as the number of simultaneously edited loci increases [106]. In addition, off-target binding remains a significant obstacle for guide design that scales with the number of targeted sites. Pangenome references are valuable tools for improving guide design as they can identify all potential off-target sites in a given population, which is necessary for cultivar-specific design of sgRNAs where variability may be present in the target sequence or protospacer adjacent motif (PAM) site within the population.

The use of pangenomes and associated knowledge of the gene content of the individual being modified can increase the reliability of genome editing technologies. The sequence of the CRISPR single guide RNA (sgRNA) is designed to match target sequences in the genome, within a specific distance up or downstream of a PAM site, which serves as the binding site for the Cas protein. The efficiency of the CRISPR-Cas system is impacted by the selection of the CRISPR target site that guides the Cas protein to a specific region within the genome of the individual. Potentially deleterious off-target activity can occur in regions of the genome that share sequence identity with the target site such as duplicated/repeated sequences [107]. Off-target effects are often undesirable and have been observed in rice (*Oryza sativa*) [108], grapevine (*Vitis vinifera*) [109], and cotton (*Gossypium*) [110]. Avoiding off-target effects requires detailed genomic information for the individual being modified [107]. Using pangenome references containing all variant data can help to avoid off-target effects because the gene editing design process will incorporate all available data and not just the data of the reference individual [111]. This comprehensive availability of data allows researchers to design specific sgRNAs that can accurately and precisely target the region of the gene (allele) and avoid mismatches due to sequence variation [12,17]. By targeting specific differences in allele sequences such as PAV and SNPs discovered through pangenomes, functional traits of crop species may be altered with great efficiency.

## 6. Future Applications of Pangenomes in Genome Editing through Super-Pangenome Guided CRISPR-Cas

A valuable target for genome editing is reintroducing agronomically beneficial genes that are lost in domesticated crop species but conserved in wild relatives. Genes can be lost in cultivars compared to wild types if they are selected against during domestication and breeding, both intentionally and unintentionally [20,112]. These lost genes can have agronomically beneficial functions such as disease resistance [113,114] or adaptations to extreme environments such as heat and drought tolerance or efficient nutrient use strategies [115]. In many crop species, genomic regions and functions unrelated to yield have been lost due to domestication selection including the Yr36 gene for rust resistance in bread wheat [116] and disease resistance genes from rice [117] and sorghum (*Sorghum bicolor*) [118]. Reintroduction of variable genes through wild introgression is possible as shown by the discovery of the TomLoxC promoter allele linked with flavour reintroduced into modern tomato cultivars [35]. However, wild introgression can potentially introduce deleterious alleles such as altered flowering time or reduced plant size [13,119,120]. Genome editing through CRISPR-Cas could allow the reintroduction of these traits without associated deleterious alleles by multiplexed editing of SVs linked to traits, but this requires thorough analysis of a wide gene pool of the species to increase specificity of the target and avoid off-target effects.

A super-pangenome aims to represent the genetic architecture of a group of taxa above the species level by combining different pangenomes from all species within that group [121]. By studying super-pangenomes, markers associated with desirable traits in wild relatives can be incorporated in domesticated cultivars. Mapping these gene PAVs through pangenomes allows for them to be compared with wild and exotic relatives to characterise advantageous traits that may have been lost as a result of selective breeding. Through CRISPR-Cas modification of targeted alleles based on wild relatives, these advantageous traits can be reintroduced in crops without also introducing associated deleterious alleles [122].

Super-pangenomes also show potential use in the future domestication of wild crop relatives as new food sources and improvement of modern crop varieties, primarily by linking PAVs and SVs to candidate domestication genes to guide CRISPR-Cas modification in a different species [121]. A benefit super-pangenomes provide for de novo domestication is a shared reference for direct comparison between all types of genomic variants present between advanced crops and wild species, in both core and dispensable regions. This allows inquiry into evolutionary divergence at loci of agronomic interest that has taken place since speciation, or over the course of domestication, highlighting specific targets for modification that may not be present at a single species level. Candidate domestication genes are genes strongly linked to traits such as increased yield, seed shatter resistance, flowering time, plant architecture, and climatic tolerance [13,123,124,125,126,127,128,129]. Modification of domestication genes through multiplexed CRISPR-Cas has been successful in wild crop relatives of tomato to produce new lines with increased fruit size, yield, and nutritional value, and greater abiotic and biotic stress tolerance than cultivated tomato lines [15,130,131,132]. Potential wild crop relatives for domestication through CRISPR-Cas include pennycress (*Thlaspi arvense*) to an oilseed crop with cold tolerance [133]; weeping grass (*Microlaena stipoides*) to a cereal crop with abiotic stress tolerance [134]; and common wild rice (*Oryza rufipogon*), wild emmer wheat (*Triticum dicoccoides*), and teosinte (*Zea mays ssp. parviglumis*) to new cereal crops with relatively high genetic diversity [135,136,137]. Furthermore, the transfer of traits between modern varieties is also possible, such as CRISPR-Cas modification of disease resistance genes in Brassica to other Brassicaceae species [138] or transfer of disease resistance genes in rust-resistant wheat varieties to other Poaceae species such as barley or sorghum [116]. Domestication of wild plants or improvement of modern crops based on candidate domestication genes in related species or genera would be a significant step in securing future food resources, as this will lead to varieties that are adapted to stressful environments or ecological niches [139], and the overall expansion of genetic diversity within agricultural systems [140,141].

Another application for super-pangenomes combined with CRISPR-Cas modification is the transfer of valuable adaptations for adverse environments across different plant genera or families through higher level super-pangenomes. While few pangenomes have been published above the species level, a super-pangenome of 10 different Poplar species was constructed [142,143] as well as a banana super-pangenome made of 15 different accessions [144]. Rapid advances in pangenome construction suggest that pangenomes spanning multiple eukaryotic genera and even families will become available in the near future. Analysis of desirable traits in genera and families related to crop species will allow multiplexed CRISPR-Cas systems to be designed to translate similar traits into crop varieties. Such adaptations could include increased photosynthetic efficiency in C3 plants such as rice via transporter adaptations of the carbon assimilation pathway in C4 plants [13,145], increased resilience to climate change based on SNPs across species associated with precipitation and thermal variability tolerance [146], and higher nutrient-use-efficiency based on phosphorus-saving strategies of the non-mycotrophic Proteaceae family in south-west Australia [147]. Development of new crop varieties with these traits through multiplexed CRISPR-Cas using super-pangenomics will be an important step to securing food resources against global food challenges such as climate change and new diseases [148].

## 7. Challenges and Considerations

A major limiting factor for the utilisation of pangenomes to guide CRISPR-Cas editing in crops is the size and number of parallel targets. The most common window sizes for base editors ranges from 4–5 bp to 50–150 bp, allowing for punctual modifications in the target [149,150,151]. Nonetheless, larger cassette insertions have been reported in plants through the use of different CRISPR methods such as non-homologous end joining (NHEJ) that have allowed insertions and deletions that are a few kilobases in size [152,153,154]. However, efficiency drops steeply as the size of the edit increases [155]. This drop in efficiency poses a significant constraint for editing SVs identified in pangenomes. Further approaches involve the development of novel Cas proteins requiring different or flexible PAM sequence sites that allow the targeting of a wider range of genomic sites, particularly when combined with multiplexed CRISPR-Cas [156]. Given the significant effect SVs have in crop evolution and diversity, future efforts should focus on developing methods to increase the size of the catalytic window for CRISPR-Cas editing to fully exploit advances in pangenomics.

With regards to shortcomings in pangenomics, iterative and de novo constructed pangenomes usually leave the chromosomal placement unspecified for variable regions that are not present in the original reference. These additional contigs can still theoretically be targeted with CRISPR for individuals in which they are present, but not knowing their genomic context can limit our understanding of regulatory elements that may influence their expression. Variation graph approaches overcome this limitation, however, they rely heavily on long-reads or deep-sequencing a large number of individuals to capture SVs at a high fidelity. Therefore, improvements in sequencing technology that allow the affordable identification of SVs are needed to enhance the information that pangenomes can provide for editing with CRISPR-Cas.

## 8. Conclusions

Pangenomes are valuable tools to identify agronomically important gene variants. To date, many plant pangenomes have been used to aid CRISPR studies in locating and targeting genomic regions of interest, broadening our understanding of QTL regions, supporting high throughput mutagenesis approaches, and linking important SVs to traits. Pangenomes have given way to a deeper understanding of functional traits that have been lost or were previously unknown in crop species. The study of pangenomics has also been used to reveal traits in wild crop species that can potentially be integrated into new or existing crops. Knowledge gained from pangenomic studies has already increased the effectiveness of the CRISPR-Cas tool to develop highly specific CRISPR target sequences, allowing for precise alteration of genome content and gene expression. While use of CRISPR-Cas systems guided by pangenomic studies is still limited by the size of catalytic windows needed for modification and the scale of SVs and PAVs linked to specific traits, pangenome construction technology is steadily advancing along with improvements in the accuracy of genome sequencing, annotation, and affordability of these processes. In the future, pangenomes will be key for thorough and effective design of genome editing experiments in crop varieties to increase global food security and resilience to climate change.

## Figures and Tables

**Table 1:** A summary of plant pangenome studies.

Species Name	Accessions	Size (Individuals)	Analysis	Reference
*Glycine max*	USDA collection	1110	PAV, GO, SNP discovery and population genetics analysis	[68]
	Chinese population	26	Synteny, SV, genetic variation and gene expression analysis	[50]
*Zea mays*	USDA Collection	14,129	GBS tagging, GWAS mapping, and PAV analysis	[51]
*Solanum lycopersicum*	NCBI SRA database	725	SNP calling, QTL mapping, expression analysis, and PAV analysis	[35]
*Brassica oleracea*	Chinese Kale/TO100	10	Gene clustering, TE annotation, SNP calling, phylogenetic, PAV, and GO analysis	[39]
*Brassica napus*	Diversity set	8	Phylogenetic, SNP, InDels, SV, PAV, population analysis	[27]
	Diversity set	53	Candidate identification, QTL, and SNP analysis	[52]
*Brassica distachyon*	Diversity set	54	Pan-gene clustering, variant calling, TE, and indel phylogenetic analysis	[53]
*Hordeum vulgare*	Diversity set	20	GWAS, inversion calling, SNP calling, QTL mapping, and PAV analysis	[54]
*Oryza sativa*	China National Rice Research Institute andNational Institute of Genetics in Japan	1529	Evolutionarily and PAV analysis	[55]
*Cajanus cajan*	ICRISAT	89	SNP and PAV analysis	[29]
	Diversity set	3366	SNP, SV, CNV, phylogenetic, GWAS analysis, and genomeprediction	[56]
*Malus domestica*	Plant Genetic Resources Unit	91	Gene prediction, comparative analysis, and PAV/variant calling	[57]
*Sesamum indicum*	Diversity set	5	PAV and evolutionary analysis	[58]
*Helianthus annuus*	USDA Collection	493	SNP calling, genome positioning, and GO and GWAS analysis	[59]
*Manihot esculenta*	Diversity set	57	Haplotype sampling and genomic prediction	[60]
*Sorghum bicolor*	Diversity set	354	PAV, SNP, GWAS, diversity, and population analysis	[36]
	Chibas sorghum breeding program	24	Genotype prediction, haplotype sampling, and WGS	[61]
*Triticum aestivum*	Chinese Spring	18	PAV and SNP analysis	[62]
*Arabidopsis thaliana*	MPI for Plant Breeding Research	7	Pangenomic, CNV, and synteny analysis	[63]
*Amborella trichopoda*	*Ambroella* Genome Project	10	PAV, GO, candidate gene, phylogenetic, and SNP analysis	[64]
*Medicago truncatula*	Diversity set	15	Comparative genomic analysis, protein orthlog, diversity, and SV analysis	[66]
*Gossypium*	NCBI database	1961	InDel, population structure, LD, SV, CNV, PAV, and metagenome association analysis	[65]
*Capsicum*	Diversity set	5	PAV and GWAS analysis	[25]
